# Allograft Injury Following Nandrolone and High‐Dose Dietary Supplements Use in a Liver Transplant Recipient: A Case Report

**DOI:** 10.1002/ccr3.70721

**Published:** 2025-07-29

**Authors:** Mahnaz Sadat Hosseini, Simin Dashti‐Khavidaki, Monavar Talebian, Mohsen Nasiri‐Toosi, Masoomeh Safaei

**Affiliations:** ^1^ Resident of Pharmacotherapy, Department of Clinical Pharmacy, Faculty of Pharmacy Tehran University of Medical Sciences Tehran Iran; ^2^ Liver Transplantation Research Center Tehran University of Medical Sciences Tehran Iran; ^3^ Department of Clinical Pharmacy, Faculty of Pharmacy Tehran University of Medical Sciences Tehran Iran; ^4^ Department of Pathology, Cancer Institute, Imam Khomeini Hospital Complex Tehran University of Medical Sciences Tehran Iran

**Keywords:** dietary supplements, drug‐induced liver injury, hepatotoxicity, herb, liver transplantation

## Abstract

This case report highlights the importance of meticulous monitoring of all medications and dietary supplements used by solid organ transplant recipients. Although the recommended daily allowances of vitamins, minerals, and herbs are generally considered safe, combining various supplement formulations can result in excessive intake, thereby increasing the risk of allograft injury in this patient population.

## 1 Introduction

Drug‐induced liver injury (DILI) denotes an acute or chronic hepatotoxicity resulting from medications and herbs or dietary supplements (HDS) [1]. Herb‐induced liver injury (HILI) is another term used to describe liver damage attributed to the use of herbal products [2]. The prevalence of DILI varies globally, with Asia reporting the highest rate of 17.82 per 100,000 individuals [3]. Three primary mechanisms of DILI are direct intrinsic hepatotoxicity, which is dose‐dependent and predictable; idiosyncratic reactions, which are dose and duration‐independent and less predictable; and indirect DILI, in which the biological action of the drug impacts the host immune system, causing liver injury that is delayed in onset and dose‐independent [1]. DILI/HILI may manifest as hepatocellular, cholestatic, or mixed‐type danages [1]. Approximately 10% of hepatotoxicity cases stem from direct drug toxicity or toxic metabolites generated during drug metabolism in the liver. Timely diagnosis and appropriate management of DILI/HILI are crucial for preserving liver function and avoiding the need for liver transplantation [1]. This case report explores the challenges associated with the indiscriminate use and self‐administration of nandrolone and dietary supplements in a liver transplant recipient.

## 2 Case History/Examination

A 37‐year‐old male, who underwent liver transplantation 7 years ago due to Wilson's disease, was referred to the pharmacotherapy clinic at Imam Khomeini Hospital Complex (IKHC) affiliated to Tehran University of Medical Sciences, Tehran, Iran, in March 2024. He had received the allograft from an unrelated deceased donor following a car accident. The patient's recent medication history included immunosuppressive therapy with cyclosporine 150 mg twice daily and mycophenolate mofetil 1000 mg twice daily that had been increased after the suggestion of acute allograft rejection in December 2023, as well as amitriptyline 60 mg daily, fluoxetine 40 mg daily, and oxazepam 30 mg daily, that had been prescribed by healthcare providers and taken for over 5 years. After several minutes of counseling, the patient declared that he was self‐administering injectable nandrolone decanoate 25 mg every 2 weeks and weekly injections of biotin, dexpanthenol, and a vitamin B1, B6, and B12 complex ampule. In addition, he was simultaneously taking various multi‐ingredient, oral dietary supplements daily for health and cosmetic purposes, such as muscle and hair strengthening. All these supplements were initiated concurrently within the past 5 months. The details of these supplements and their components are presented in Table 1.

**TABLE 1:** The components of each supplement consumed by the patient and the patient's daily intake.

The components of each supplement	Hairtamin advanced	Vitally tone	Pharmaton	Elman	Wellman	Hair‐vit	Daily intake of the patient
Vitamin A and Beta‐Carotene	500 IU	4000 IU	4000 IU		2500 IU		11,000 IU
Vitamin D	18 IU	400 IU	400 IU	—	200 IU	—	1018 IU
Vitamin E	—	18 IU	15 IU	20 IU	30 IU	—	83 IU
Vitamin C	50 mg	80 mg	60 mg	60 mg	60 mg	—	310 mg
Vitamin B1	2.5 mg	2 mg	2 mg	12 mg	12 mg	10 mg	40.5 mg
Vitamin B2	2.5 mg	2.4 mg	2 mg	12 mg	5 mg	10 mg	33.9 mg
Vitamin B3	2.5 mg	16 mg	15 mg	18 mg	20 mg	20 mg	91.5 mg
Vitamin B6	2.5 mg	1.4 mg	1 mg	20 mg	9 mg	5 mg	38.9 mg
Vitamin B12	10 mcg	2.5 mcg	1 mcg	4 mcg	9 mcg	2 mcg	28.5 mcg
Biotin	6000 mcg	—	—	150 mcg	50 mg	20 mcg	6220 mcg
Panthotenic acid/calcium pantothenate	10 mg	9.16 mg	20 mg	18 mg	10 mg	10 mg	77.16 mg
Folic acid	400 mcg			400 mcg	500 mcg		1300mcg
Iron		14 mg	10 mg	18 mg	6 mg	12 mg	60 mg
Magnesium		100 mg	10 mg	70 mg	50 mg		230 mg
Zinc	15 mg	10 mg	1 mg	20 mg	15 mg	8 mg	69 mg
Iodine					150 mcg		150 mcg
Manganese		2 mg	1 mg		3 mg		6 mg
Copper		1 mg	1 mg		1 mg		3 mg
Chromium					50 mcg		50 mcg
Selenium	70 mcg			20 mcg	150 mcg		240 mcg
Silicon					10 mg		10 mg
Calcium	100 mg		90.3 mg	200 mg			390.3 mg
Phosphorus	77 mg		70 mg				147 mg
Fluoride	0.2 mg		0.2 mg				0.4 mg
Potassium	4.2 mg		8 mg				12.2 mg
Co‐enzyme Q10					2 mg		2 mg
Arginine					20 mg		20 mg
Methionine				60 mg	20 mg	30 mg	110 mg
Cysteine				60 mg		30 mg	90 mg
L‐carnitine					30 mg		30 mg
Para‐ aminobenzoic acid				100 mg	20 mg	10 mg	130 mg
Choline and derivatives, Linoleic acid, Linolenic acid, Inositol	26 mg		92 mg	200 mg		100 mg	418 mg
Yeast						100 mg	100 mg
Hydrolyzed gelatine powder						50 mg	50 mg
Ginseng extract	40 mg		40 mg		20 mg		100 mg
Bioflavonoids					10 mg		10 mg
Rutin powder	20 mg		20 mg				40 mg
Soy lecithin	66 mg					20 mg	86 mg
‐ *Aloe vera* leaf powder ‐ *Bacopa monnieri* leaf extract ‐Rosemary leaf powder ‐*Stinging nettle* root Powder ‐Lutein 5% ‐Horsetail herb extract ‐Turmeric root extract (95% curcumin) ‐Cayenne fruit powder ‐Black pepper seed extract (95% piperine)	23.7 mg						23.7 mg

## 3 Differential Diagnosis, Investigation and Treatment

In December 2023, the patient had been admitted to the liver transplant ward of IKHC with elevated liver enzymes: aspartate aminotransferase (AST) from baseline 21 to 290 IU/L (institution laboratory range: 10–50 IU/L), alanine aminotransferase (ALT) from baseline 23 to 210 IU/L (institution laboratory range: 10–40 IU/L), alkaline phosphatase (ALP) from baseline 185 to 961 IU/L (institution laboratory range: 70–460 IU/L), and total bilirubin (T Bill) from baseline 0.7 to 2.5 mg/dL (institution laboratory range: 0.1–1.2 mg/dL). International normalized ratio (INR) remained stable at 1.0. His maintenance immunosuppression regimen within the past several months consisted of cyclosporine 100 mg twice daily (trough level 74 ng/mL) and mycophenolate mofetil 500 mg twice daily that were appropriately dosed for age, weight, and time since liver transplantation (7 years post‐procedure). No prior episodes of allograft rejection or biopsy‐proven injury were documented.

**FIGURE 1:**
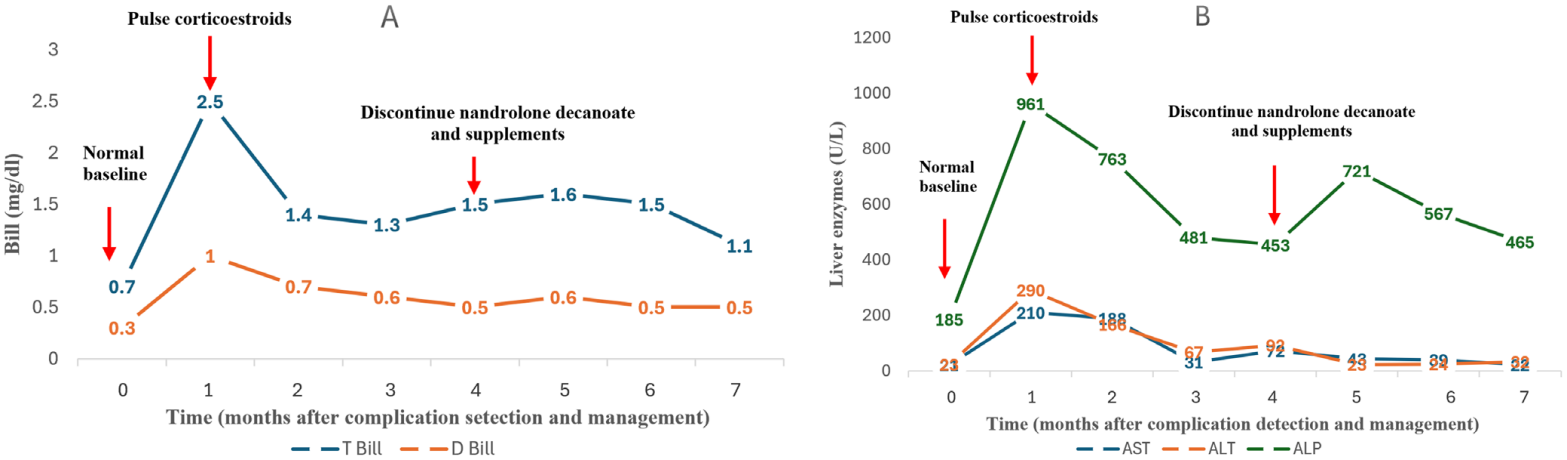
The diagram of patient's liver enzymes. (A) shows a decreasing trend of total and direct bilirubin, and (B) presents a transient decreasing trend of aminotransferases and alkaline phosphatase after corticosteroid pulse therapy and an increasing trend afterward. Following the discontinuation of nandrolone decanoate and dietary supplements, liver enzymes decreased to normal value and remained normal during 3 months of follow‐up. ALP, Alkaline phosphatase; ALT, Alanine aminotransferase; AST, Aspartate transferase; D Bill, Direct bilirubin; T Bill, Total bilirubin.

**FIGURE 2:**
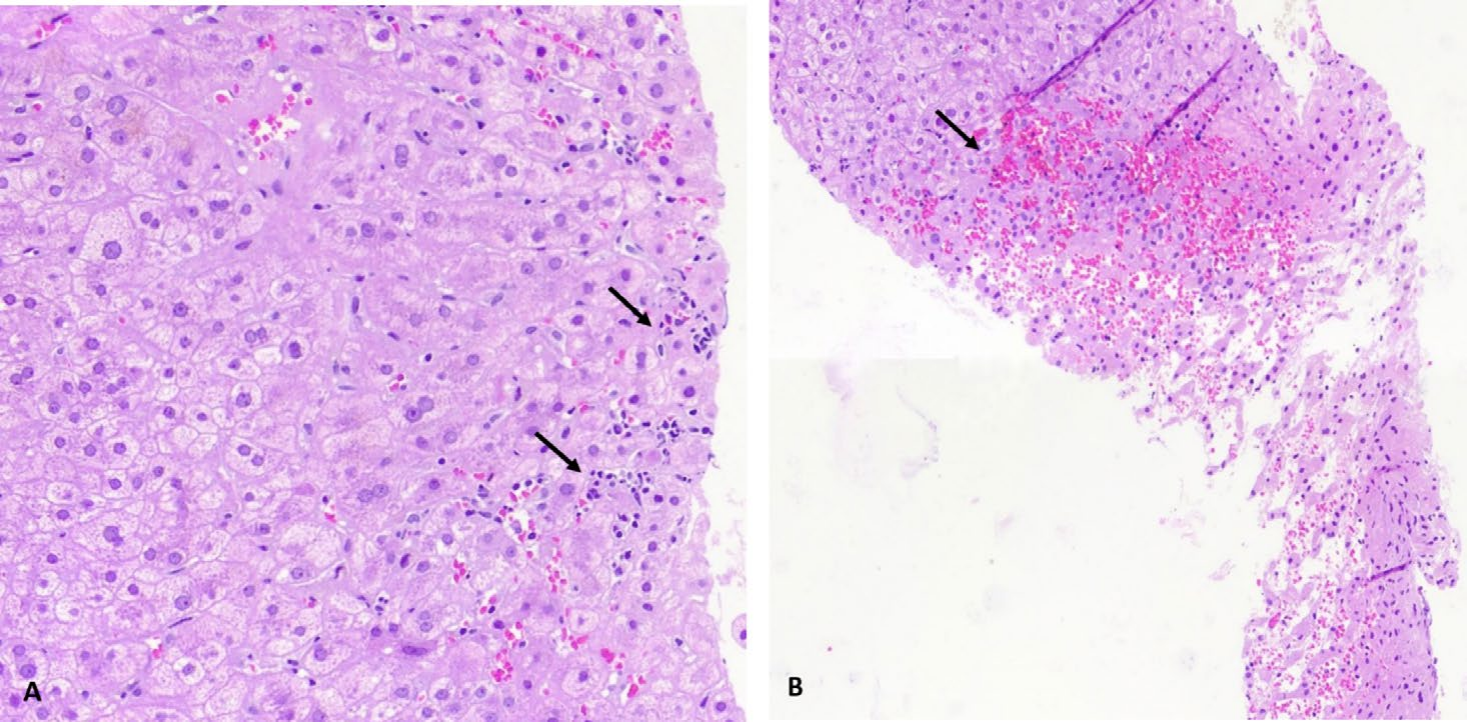
Liver biopsy findings. Microscopic liver examination in (A) reveals mild lobular inflammation (arrows) (×400), and in (B) focal congestion (arrow) is noted (×100).

During hospital admission, diagnostic evaluation included viral hepatitis serologies (HAV, HBV, HCV, CMV, EBV), autoimmune markers (antinuclear, anti‐smooth muscle, and antimitochondrial antibodies), iron studies (ferritin, transferrin saturation), thyroid function tests, and abdominal ultrasound. All serologic test results returned normal. No recent history of hypotension, sepsis, heart failure, or other conditions that are risk factors for ischemic liver injury were identified. The patient denied alcohol use. Abdominal ultrasound revealed no dilation in the common and intrahepatic bile ducts and a normal diameter of the portal vein. Ultrasonic color Doppler imaging of the portal and splenic veins exhibited normal hepatopetal flow in the portal vein, along with normal parameters and flow in the splenic vein, portal vein, and collaterals. Concerns for acute rejection arose due to self‐reported recent non‐adherence to immunosuppressive therapy. Liver biopsy was performed. Pending biopsy results, empiric therapy was initiated with intravenous methylprednisolone 1 g every other day for 3 doses and immunosuppression intensification with an increasing cyclosporine dose to 150 mg twice daily and mycophenolate mofetil to 1000 mg twice daily. After methylprednisolone pulse therapy, oral prednisolone was started at a daily dose of 50 mg and subsequent rapid tapering. Antimicrobial prophylaxis with cotrimoxazole 800/160 mg daily for 3 months and fluconazole 150 mg daily for 10 days were started. This therapeutic approach followed institutional protocols for suspected acute rejection while awaiting histopathological confirmation. Laboratory trends of liver function tests are shown in Figure 1. The liver biopsy findings indicated the presence of eight portal areas in the specimen, characterized by normal connective tissue stroma without fibrosis, absence of significant inflammation or interface damage in portal areas, intact bile ducts, no copper deposits, no steatosis, and no bilirubin stasis. Mild intracellular edema and occasional Mallory bodies were observed in some hepatocytes. Focal aggregation of a small number of lymphocytes in certain areas associated with lytic necrosis was noted, along with the presence of rare apoptotic bodies. Confluent necrosis was not identified. The hepatic cords and reticulin framework were within normal parameters. Dilatation and congestion of some hepatic sinusoids, primarily in zone 3, were observed, while other vascular channels appeared unaltered. No significant C4d deposits were detected on the endothelial cells of the portal and central venules. The pathologist's assessment indicated mild lobular necroinflammatory damage, absence of histologic signs of acute or chronic rejection, and a suspected likelihood of medication‐induced injury (Figure 2).

## 4 Conclusion and Results

Due to suspicion of medication‐induced injury, after hospital discharge the patient was visited in the transplant pharmacotherapy clinic. The clinical transplant pharmacist identified the use of nandrolone decanoate and several parenteral and oral HDS by the patient as detailed above and in Table 1. This history had not been disclosed by the patient at the time of hospital admission. Table 2 represents the different types of liver damage that have been reported in the literature with nandrolone and HDS the patient used. No data regarding DILI/HILI was found for other components of consumed HDS.

**TABLE 2:** Potential medicine, minerals, vitamins, and herbals induced hepatotoxicity.

Components	Drug ‐herb interaction	Hepatocellular injury	Cholestatic injury	Chronic hepatitis	Cirrhosis	Liver adenoma	Sinusoidal obstruction syndrome/Peliosis hepatis	Fibrosis and necrosis
Nandrolone decanoate		√	√			√	√	
Vitamin A and Beta Caroten		√	√	√	√			
Iron		√			√			√
Zinc		√			√			√
Manganese								√
Copper		√						√
Selenium								√
Chromium		√						√
Ginseng	√							
*Aloe vera*		√						
Horsetail		√						
Turmeric	√	√						

The R value defined as serum ALT/upper limit of normal (ULN) divided by serum ALP/ULN using laboratory tests at the time of hospital admission was calculated to be 2.625, which suggests mixed hepatocellular and cholestatic liver injury. The Roussel‐Uclaf Causality Assessment Method (RUCAM), 2016 version [2], was applied to evaluate the causality between the patient's liver injury and the various medications and HDS he was taking, as listed in Table 2. RUCAM scores of 6 and 8 were obtained for HDS and nandrolone, respectively, indicating a probable association with DILI/HILI in this patient.

The patient agreed to discontinue nandrolone decanoate and supplements. During the three‐month follow‐up after the visit to the transplant pharmacotherapy clinic, serum concentrations of transaminases, alkaline phosphatase, and bilirubin became normalized (Figure 1).

## 5 Discussion

The liver is highly vulnerable to damage from various insults, including viral infections, autoimmune disorders, metabolic abnormalities, and exposure to toxic substances such as medications HDS. DILI or HILI can result from a wide range of agents, including anabolic androgenic steroids, vitamins, minerals, and herbal products [1]. HDS are responsible for approximately 20% of all cases of adult liver injury [4]. As seen in Table 1, our patient had been taking nandrolone decanoate at a monthly dosage of 50 mg and consuming 11,000 units of vitamin A on a daily basis. Furthermore, the patient exceeded the daily recommended intake levels of several minerals, including iron, zinc, manganese, copper, selenium, and chromium, by consuming a variety of supplements. These supplements also contained herbal components such as ginseng, turmeric, horsetail, and *Aloe vera* , which are known to pose risks for herb‐drug interactions. Importantly, the safety profile of these herbal products has not been thoroughly evaluated in liver transplant recipients. DILI/HILI typically occurs within 6 months of initiating the offending agent [1], a timeline consistent with the clinical presentation observed in this patient. Table 2 summarizes the types of liver damage reported with the agents identified in this case. However, no reports were found regarding the hepatotoxicity or modulation of cytochrome P450 enzymes for the other medications, vitamins, minerals, and herbs the patient was taking, as shown in Table 1 but not listed in Table 2. The concomitant use of multiple potentially hepatotoxic agents (Table 2) may have increased the risk of DILI/HILI in this patient. Given that DILI is primarily a clinical diagnosis of exclusion [1], comprehensive viral, autoimmune, imaging, and histologic evaluations were performed to rule out other causes of liver damage.

Nandrolone decanoate, a synthetic anabolic androgenic steroid, is commonly misused to enhance performance, augment lean body mass, and improve muscle fiber quality [5]. The induction of liver damage by anabolic androgenic steroids can be ascribed to disruptions in mitochondrial respiratory chain function, accumulation of reactive oxygen species and oxidative stress, and heightened infiltration of inflammatory cells into the hepatic tissue. These conditions may also trigger activation of stellate cells in the liver and promote excessive collagen deposition in the liver parenchyma [6]. Liver injury resulting from anabolic‐androgenic steroid use typically manifests within 1 to 4 months of initiation, although delayed onset, occurring between 6 to 24 months, has also been reported [5].

Vitamins are essential dietary elements that are generally not synthesized by the human body. Fat‐soluble vitamins, which are stored in the liver, have the potential to induce liver damage in excessive quantities. Vitamin A is a vital vitamin for vision, skin health, bone strength, and immunity and is typically recommended at a daily intake of approximately 700–900 mcg for adults [7]. Elevated levels of vitamin A can accumulate in hepatic stellate cells, leading to cellular hypertrophy, increased collagen production, fibrosis, and ultimately, liver impairment. Clinically, this may manifest as elevated serum bilirubin, aminotransferases, and alkaline phosphatase, as well as the development of portal hypertension with associated ascites and esophageal varices. The onset of liver damage due to vitamin A excess can occur over a period of weeks to months [8].

Minerals play a pivotal role in maintaining overall health, contributing to various physiological functions such as skeletal health, neuromuscular function, and metabolic processes. The recommended dietary intake levels for essential minerals like iron, zinc, manganese, copper, selenium, and chromium in adult males are typically set at 8–11 mg, 15 mg, 2.3 mg, 1.5 mg, 55 mcg, and 35 mcg, respectively [9]. Overconsumption of minerals can directly impact liver function, leading to potential toxicity. Excessive accumulation of minerals in the liver can result in fibrosis and necrosis [10].

Herbal supplements have the potential to induce liver damage. Ginseng, a renowned herbal remedy derived from the root of the perennial plant *Panax ginseng* , has not been directly linked to liver injury. However, it is known to inhibit the cytochrome P450 (CYP) 3A4 isoenzyme, impacting the metabolism of drugs such as calcineurin inhibitors like cyclosporine, which was part of our patient's medication regimen [11]. Ginseng also contains pyrrolizidine alkaloids [12] which have been reported to induce sinusoidal congestion with hepatocyte necrosis [1]. Notably, our patient's liver biopsy exhibited these specific histologic changes, suggesting a potential contribution of ginseng to the observed liver injury. Turmeric, derived from the root of the *Curcuma longa* plant, is a popular herbal product that has been associated with hepatocellular damage. Liver injury from turmeric and its active component curcumin can present any time from a few weeks to several months after intake [13]. Curcumin, a constituent of turmeric, may inhibit CYP1A2, CYP2B6, CYP2C9, CYP2D6, and CYP3A4 isoenzymes, consequently altering the metabolism of drugs, notably cyclosporine [14]. *Aloe vera* , when consumed orally, can result in hepatocellular injury with a clinical course resembling acute viral hepatitis. The toxicity induced by oral *Aloe vera* may take several months to become apparent [15]. Horsetail, an extract sourced from the *Equisetum arvense* plant, has been linked to acute hepatocellular injury and jaundice, presenting around 2 weeks after administration [16].

Based on histological analysis of the patient's liver biopsy, there was a strong suspicion of toxin‐ or drug‐induced liver injury. The liver injury phenotype in this patient—characterized by elevated ALT, AST, ALP, and total bilirubin, along with necroinflammatory changes and sinusoidal congestion on histology—aligns with the direct hepatotoxicity patterns described in the American Association for the Study of Liver Diseases (AASLD) guidance [1]. Considering the patient's use of nandrolone decanoate alongside multiple supplements containing vitamins and herbal components with potential hepatotoxicity, and applying the RUCAM causality assessment method, probable DILI/HILI was considered in this patient.

The main challenge in this patient was the concurrent use of nandrolone, alongside several herbs and dietary supplements often in high doses over the same timeline compatible with inducing DILI/HILI. Furthermore, the compositional complexity and potential for inaccurate labeling of dietary supplements complicate precise causality assessment; however, necroinflammatory findings on the patient's liver biopsy and sinusoidal congestion without evidence of allograft rejection, biliary cholestasis, or adenoma may be due to side effects of high doses of vitamin A, high doses of different minerals, nandrolone decanoate, and potentially ginseng.

The primary and most effective approach in managing DILI involves halting the use of the causative agent [17]. In some instances, corticosteroids are employed for the treatment of DILI [1, 17, 18]. Initially, prior to receiving the liver biopsy results and while unaware of the patient's use of nandrolone and HDS, the healthcare team treated the patient with a methylprednisolone pulse followed by oral prednisolone, based on a suspicion of acute allograft rejection. During hospitalization, the patient did not use nandrolone decanoate or any supplements, and liver enzyme levels decreased at that time. However, upon discharge, the reintroduction of these substances by the patient led to a subsequent increase in enzyme levels (Figure 1). Following referral to the transplant pharmacotherapy clinic and complete cessation of these agents, the patient's enzyme levels improved.

In conclusion, this case report underscores the significant risk of allograft injury in liver transplant recipients associated with the prolonged and often concealed use of medications and HDS. Clinical transplant pharmacists possess unique expertise in evaluating the composition and safety of these supplements in transplant patients, identifying potential interactions with immunosuppressive drugs and other medications, and determining appropriate dosages. Therefore, referral of transplant patients to specialized transplant pharmacotherapy clinics for comprehensive medication review and management is crucial for preventing drug‐induced allograft injury and optimizing clinical outcomes by mitigating the risks associated with medications, dietary supplements, and complementary alternative medicine.

## Author Contributions

**Mahnaz Sadat Hosseini:** data curation, writing – original draft, writing – review and editing. **Simin Dashti‐Khavidaki:** data curation, supervision, validation, writing – review and editing. **Monavar Talebian:** data curation, writing – review and editing. **Mohsen Nasiri‐Toosi:** writing – review and editing. **Masoomeh Safaei:** data curation, writing – review and editing.

## Disclosure

*Permission to Reproduce Material From Other Sources*: Not applicable.

## Ethics Statement

The patien's data are presented anonymously and with protecting the patient's identity.

## Consent

The patient signed a written informed consent form to publish his data anonymously.

## Conflicts of Interest

The authors declare no conflicts of interest.

## Data Availability

The authors have nothing to report.
